# High-Level Production of Isoleucine and Fusel Alcohol by Expression of the Feedback Inhibition-Insensitive Threonine Deaminase in *Saccharomyces cerevisiae*

**DOI:** 10.1128/aem.02130-21

**Published:** 2022-03-08

**Authors:** Shota Isogai, Akira Nishimura, Atsushi Kotaka, Naoyuki Murakami, Natsuki Hotta, Hiroki Ishida, Hiroshi Takagi

**Affiliations:** a Division of Biological Science, Graduate School of Science and Technology, Nara Institute of Science and Technology, 8916-5 Takayama, Ikoma, Nara 630-0192, Japan; b Research Institute, Gekkeikan Sake Co. Ltd., 101 Shimotoba-koyanagi-cho, Fushimi-ku, Kyoto 612-8385, Japan; Kyoto University

**Keywords:** *Saccharomyces cerevisiae*, sake yeast, isoleucine, threonine deaminase Ilv1, allosteric regulation, fusel alcohol

## Abstract

A variety of the yeast Saccharomyces cerevisiae with intracellular accumulation of isoleucine (Ile) would be a promising strain for developing a distinct kind of sake, a traditional Japanese alcoholic beverage, because Ile-derived volatile compounds have a great impact on the flavor and taste of fermented foods. In this study, we isolated an Ile-accumulating mutant (strain K9-I48) derived from a diploid sake yeast of S. cerevisiae by conventional mutagenesis. Strain K9-I48 carries a novel mutation in the *ILV1* gene encoding the His480Tyr variant of threonine deaminase (TD). Interestingly, the TD activity of the His480Tyr variant was markedly insensitive to feedback inhibition by Ile, but was not upregulated by valine, leading to intracellular accumulation of Ile and extracellular overproduction of 2-methyl-1-butanol, a fusel alcohol derived from Ile, in yeast cells. The present study demonstrated for the first time that the conserved histidine residue located in a linker region between two regulatory domains is involved in allosteric regulation of TD. Moreover, sake brewed with strain K9-I48 contained 2 to 3 times more 2-methyl-1-butanol and 2-methylbutyl acetate than sake brewed with the parent strain. These findings are valuable for the engineering of TD to increase the productivity of Ile and its derived fusel alcohols.

**IMPORTANCE** Fruit-like flavors of isoleucine-derived volatile compounds, 2-methyl-1-butanol (2MB) and its acetate ester, contribute to a variety of the flavors and tastes of alcoholic beverages. Besides its value as aroma components in foods and cosmetics, 2MB has attracted significant attention as second-generation biofuels. Threonine deaminase (TD) catalyzes the first step in isoleucine biosynthesis and its activity is subject to feedback inhibition by isoleucine. Here, we isolated an isoleucine-accumulating sake yeast mutant and identified a mutant gene encoding a novel variant of TD. The variant TD exhibited much less sensitivity to isoleucine, leading to higher production of 2MB as well as isoleucine than the wild-type TD. Furthermore, sake brewed with a mutant yeast expressing the variant TD contained more 2MB and its acetate ester than that brewed with the parent strain. These findings will contribute to the development of superior industrial yeast strains for high-level production of isoleucine and its related fusel alcohols.

## INTRODUCTION

**FIG 1:**
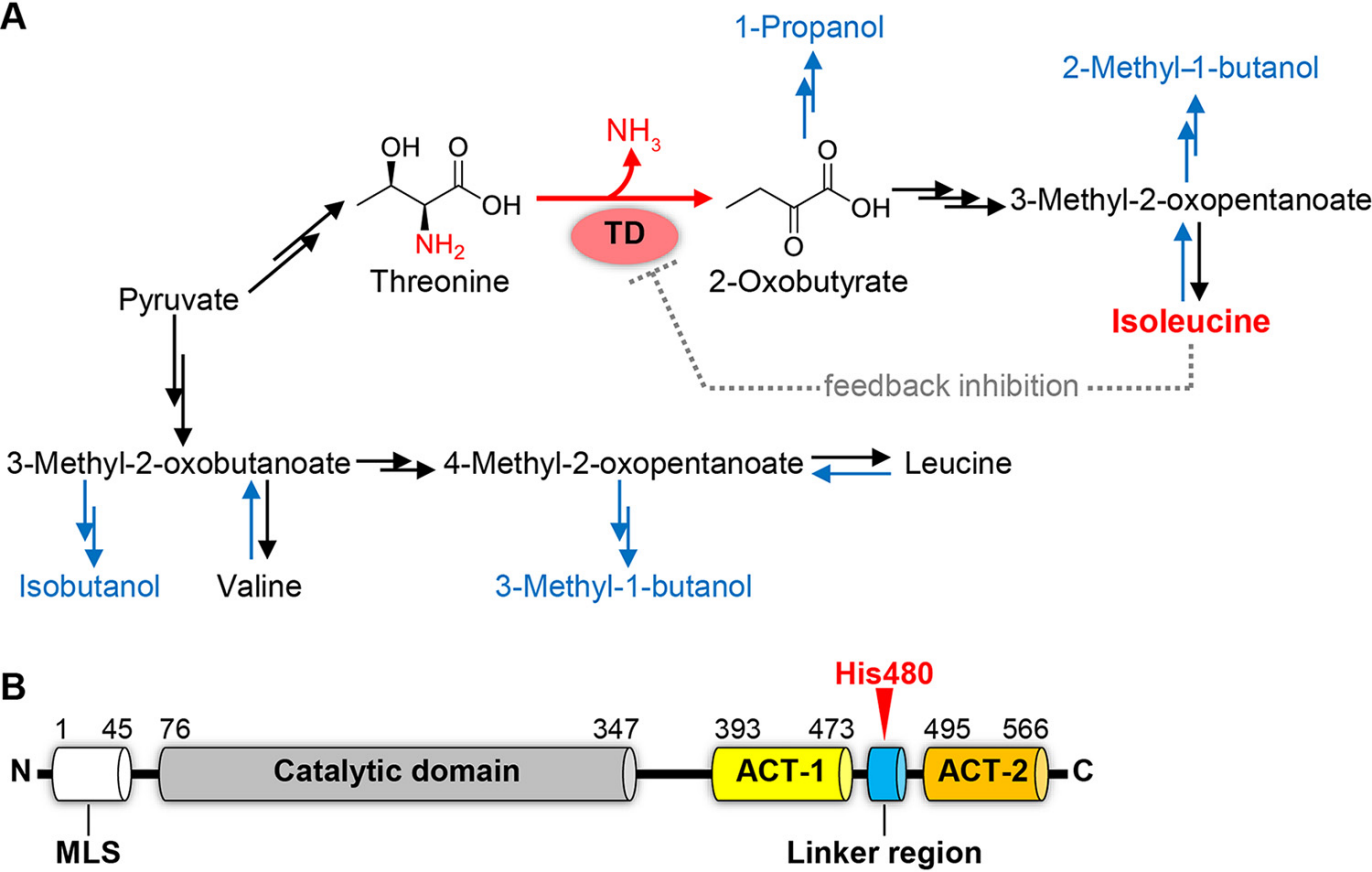
Metabolic pathways of BCAAs and domain organization of Ilv1. (A) Metabolic pathways for BCAAs and fusel alcohols. Black and blue arrows represent the biosynthetic pathways of BCAAs (isoleucine, valine, and leucine) and the catabolic pathways of BCAAs and threonine to fusel alcohols by the Ehrlich pathway, respectively. Threonine deaminase (TD) catalyzes the first and the rate-limiting step in the isoleucine biosynthesis to form 2-oxobutyrate by the deamination of threonine. The enzymatic activity of TD is subjected to feedback inhibition by the end product isoleucine. (B) Domain organization of Ilv1. Putative mitochondrion localization signal (MLS), aspartate kinase, chorismate mutase and TyrA (ACT)-like domain-1 (ACT-1), linker region between two ACT-like domains and ACT-like domain-2 (ACT-2) are represented as white, gray, yellow, blue and orange bars, respectively. His480 is indicated as a red triangle.

Sake, a traditional Japanese alcoholic beverage, is made from polished rice by multi-parallel fermentation of the fungus Aspergillus oryzae and the yeast Saccharomyces cerevisiae (1). Since sake has become popular all around the world, the development of yeast strains for the production of unique, distinctive sakes has attracted a great deal of attention. During the sake fermentation process by S. cerevisiae, threonine (Thr) and branched-chain amino acids (BCAAs; isoleucine (Ile), valine (Val) and leucine (Leu)) are catabolized by the Ehrlich pathway to form volatile compounds, known as fusel alcohols (2): 1-propanol from Thr, 2-methyl-1-butanol (2MB) from Ile, isobutanol from Val, and 3-methyl-1-butanol (3MB) from Leu (Fig. 1A). The composition of fusel alcohols and their esters have a great impact on the flavor and taste of alcoholic beverages owing to their fruit-like flavors (3, 4). For instance, 2-methylbutyl acetate has an apple-like flavor, and 3-methylbutyl acetate has a banana-like flavor; these are the acetate esters of 2MB and 3MB, respectively. Therefore, S. cerevisiae with accumulation of Ile may be valuable in producing a value-added sake because its accumulated intracellular Ile is converted into 2-MB and its acetate ester during the fermentation. In addition to their value as aroma components in foods and cosmetics, fusel alcohols including 2MB have attracted significant attention as second-generation biofuels (5). To date, several studies have performed metabolic engineering of the biosynthetic pathways of fusel alcohols for the purpose of increasing production of these compounds in microorganisms (6–11). Conventional breeding or metabolic engineering for the development of an Ile-accumulating yeast strain thus has great potential for high-level and cost-effective production of 2MB and its derivatives.

Ile is synthesized via Thr by five enzymatic steps (12, 13). Threonine deaminase (TD), also known as threonine dehydratase or threonine ammonia lyase, catalyzes the first reaction of Ile biosynthesis to form 2-oxobutyrate by the deamination of Thr using pyridroxal-5′-phosphate (PLP) as a cofactor. As shown in Fig. 1A, this deamination reaction is the rate-limiting step in Ile biosynthesis because the enzymatic activity of TD is allosterically inhibited by the end product Ile (14–19). TD therefore has been subjected to metabolic engineering for high-level production of Ile (20, 21); however, the detailed mechanisms underlying Ile-mediated allosteric regulation of TDs have not been clarified.

In S. cerevisiae, TD is encoded by the *ILV1* gene (22, 23). The amino acid sequence of Ilv1 indicates that the domain organization of Ilv1 consists of an N-terminal catalytic domain, two C-terminal regulatory domains and a linker region between the two regulatory domains (Fig. 1B). The C-terminal regulatory domains exhibit a similar secondary structure to those of Aspartate kinase, Chorismate mutase and TyrA (ACT) domain, the so called as “ACT-like” domains. The ACT domain corresponds to a regulatory ligand binding domain and is widely involved in allosteric regulation of enzymes responsible for amino acid metabolisms (24–26). The role of ACT-like domains in TDs for allosteric regulation has been well analyzed in terms of function. For instance, Tyr423 and Tyr526 in the ACT-like domains of S. cerevisiae Ilv1 are responsible for Ile recognition (19, 27). In addition, site-directed mutagenesis analyses of TDs from Arabidopsis thaliana and Escherichia coli revealed that amino acid substitutions of the residues located in ACT-like domains decreased the sensitivity to feedback inhibition by Ile (28–31). The similarity of domain organization and the conservation of these residues that are so important for Ile recognition in Ilv1 suggests that the ACT-like domains of Ilv1 are involved in the regulation by Ile, similar to those in TDs from *A. thaliana* and E. coli, such as a decrease in the sensitivity to feedback inhibition by substitution of arginine with phenylalanine at position 416 of Ilv1 (32) (Fig. S1). On the contrary, the importance of the linker region between two ACT-like domains (Blue bar in Fig. 1B) has been unclear. This region would not directly interact with effectors and residues responsible for binding to effectors (33); however, the cysteine substitution of arginine at position 499 in the linker region of the *A. thaliana* TD (Arg482 in the S. cerevisiae Ilv1) decreased the sensitivity to Ile feedback inhibition and increased Ile productivity, suggesting that this linker region is involved in allosteric regulation (29).

In this study, we isolated a sake yeast mutant with intracellular Ile accumulation (strain K9-I48). This mutant contributed to a significant increase in the contents of 2MB and its acetate ester in sake. In strain K9-I48, a heterozygous mutation was found in the *ILV1* gene corresponding to amino acid substitution of histidine with tyrosine at position 480. It was also revealed that TD activity in the His480Tyr variant Ilv1 was completely desensitized to both downregulation by Ile and upregulation by Val when these effectors were added at 1 mM for Ile and 5 mM for Val. Furthermore, the expression of the His480Tyr variant Ilv1 in the laboratory yeast increased extracellular 2MB content as well as intracellular Ile accumulation.

## RESULTS

### Isolation of sake yeast mutant with intracellular Ile.

**FIG 2:**
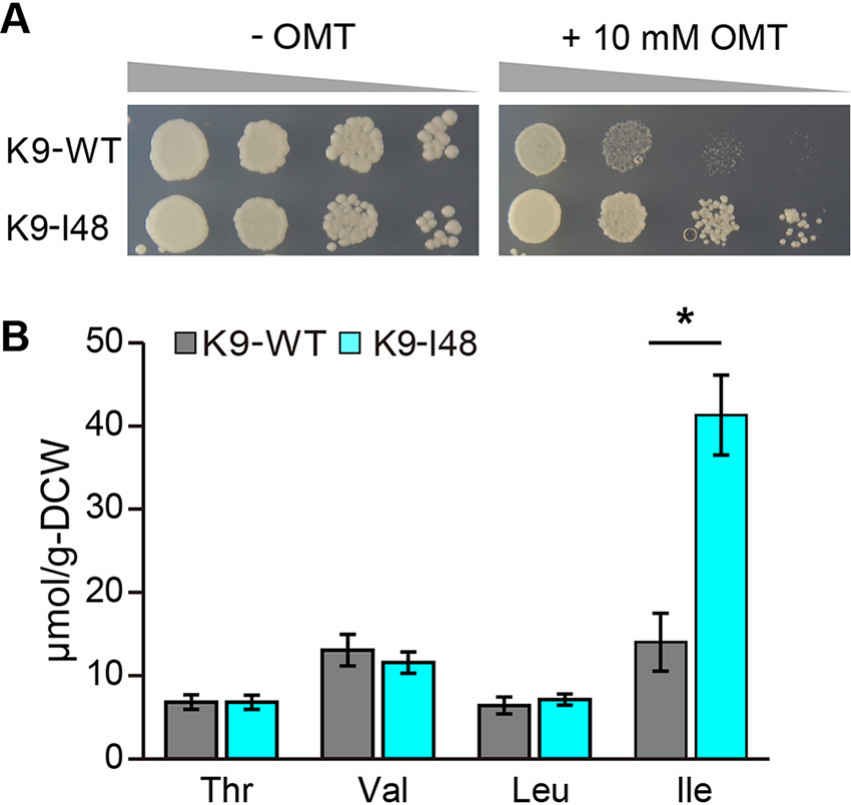
Isolation of sake yeast mutant with intracellular Ile accumulation (A) Sensitivity to OMT. Yeast cells were cultured to the early log phase and spotted on to SD+Alt without OMT (left panel) or the same medium containing 10 mM OMT (right panel). (B) Intracellular amino acids content. Strains K9-WT and K9-I48 were cultured in SD+Am medium for 48 h and intracellular amino acid contents were measured. Data are presented as means ± standard deviation from three independent experiments. Asterisks indicate statistically significant differences between two strains (Student's *t* test, *P < 0.05).

To obtain Ile-accumulating sake yeast mutants, we first isolated sake yeast mutants resistant to the isoleucine toxic analogue *O*-methyl threonine (OMT) from the parent strain K9-WT. OMT would be incorrectly used as building blocks of proteins instead of Ile due to structure similarity of OMT to Ile, which would cause dysfunction of proteins, resulting in cell death (34). Overproduction of Ile can confer the OMT resistance to cells to avoid miss-utilization of OMT. Thus, the OMT-resistant mutants are expected to synthesize large amount of Ile. When yeast cells treated with ethyl methanesulfonate (EMS) were plated on SD+Alt medium containing 10 mM OMT, approximately 100 OMT-resistant colonies were obtained. Among them, strain K9-I48 clearly exhibited OMT resistance compared to strain K9-WT (Fig. 2A) and had 2.9-fold higher intracellular Ile content (41 μmol/g-DCW) compared with that in strain K9-WT (14 μmol/g-DCW). Other BCAAs and Thr contents were almost the same in the two strains (Fig. 2B).

### Nucleotide sequence of the *ILV1* gene in strain K9-I48.

It has been reported that the amino acid substitutions in TD decreased the sensitivity of enzymatic activity to feedback inhibition by Ile, resulting in increased Ile content in E. coli and *A. thaliana* cells (29, 31). We analyzed the nucleotide sequences of the *ILV1* gene encoding TD from parent K9-WT and mutant K9-I48 yeast strains using direct PCR DNA sequencing. Interestingly, it was found that the *ILV1* gene sequence in strain K9-I48 included a heterozygous mutation, which was a mixture of cytosine and thiamin at nucleotide position 1,438, leading to one amino acid change of histidine to tyrosine at position 480 (His480Tyr) in the amino acid sequence of TD. This mutation has not been previously reported. Histidine at position 480 in the linker region is a highly conserved residue among various TDs in microorganisms and plants (Fig. S1).

### Effect of the His480Tyr substitution on TD activity of Ilv1.

**FIG 3:**
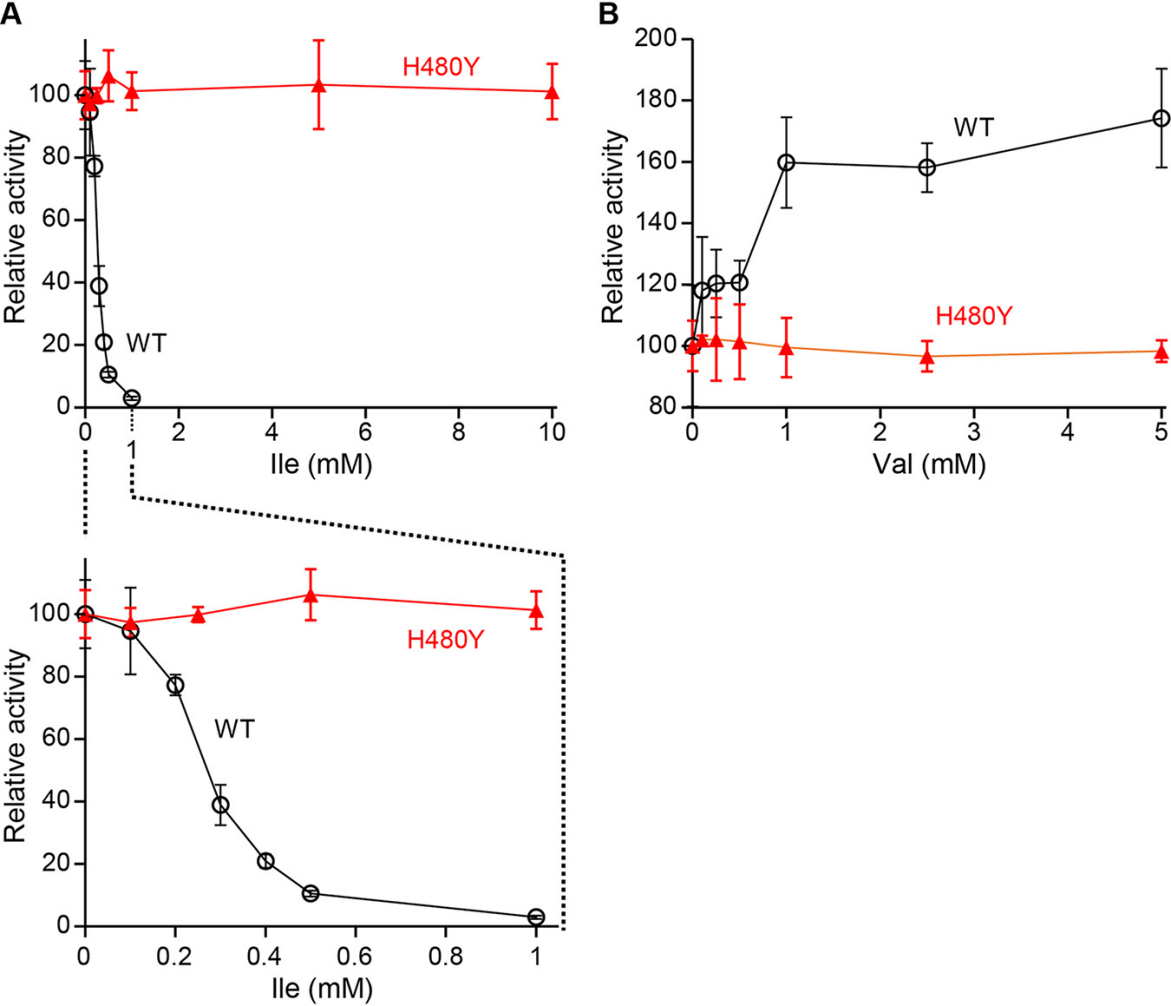
Effect of the His480Tyr substitution on allosteric regulation of TD by Ile and Val. The relative TD activities of the recombinant wild-type (WT) and H480Y variant (H480Y) Ilv1 were measured in the presence of Ile (A) and Val (B). The enzymatic activities in the absence of effectors are defined as 100% (41 U/mg for the WT Ilv1 and 57 U/mg for the H480Y variant, respectively). Data are presented as means ± standard deviation from three independent experiments. The panels in (A), upper panel represents relative activities in the presence of 0–10 mM Ile; lower panel is a zoom of the upper panel at a concentration of 0–1 mM.

In order to analyze the influence of the His480Tyr substitution on enzymatic activity of TD, the recombinant Ilv1 of the WT and His480Tyr variant (H480Y) was expressed and purified using E. coli BL21(DE3) cells. When enzymatic activity of the recombinant Ilv1 was measured in the absence of effectors, the TD activity of the H480Y variant (57 ± 5 U/mg) was slightly higher than that of the WT Ilv1 (41 ± 7 U/mg). We next analyzed feedback inhibition of the TD activity by Ile (Fig. 3A). In the presence of Ile, the TD activity of the WT Ilv1 was inhibited to 77% by 0.2 mM Ile and consequently decreased when the Ile concentration was increased. The TD activity of the WT Ilv1 was almost lost in the presence of 1.0 mM Ile. The half maximal inhibitory concentration (IC_50_) values of the WT Ilv1 was determined as 0.27 ± 0.009 mM. Given that the intracellular concentrations of Ile in yeast range from 0.4 to 0.7 mM (35, 36), these data indicate that the TD activity of S. cerevisiae cells is subject to feedback inhibition by Ile *in vivo*; that is, TD is the rate-limiting enzyme in the Ile biosynthesis of S. cerevisiae cells. In contrast, it is noteworthy that the H480Y variant Ilv1 was fully insensitive to feedback inhibition at Ile concentration up to 10 mM Ile (Fig. 3A). Ahmed et al. (1976) reported that Val upregulates TD activity (22); therefore, we also analyzed the effect of Val on the TD activity of Ilv1. As expected, the addition of Val increased the TD activity of the WT Ilv1; enzymatic activity in the presence of 5 mM Val was 1.8-fold higher than that in the absence of Val. However, TD activity of the H480Y variant Ilv1 was not upregulated by the addition of Val (Fig. 3B). Finally, we analyzed the effect of Ile on the steady-state kinetic parameters of the WT and the H480Y variant of Ilv1. As reported in previous studies (37), Ilv1 exhibited the sigmoidal kinetics (Fig. S2). Kinetic parameters, *K*_0.5_, *V*_max_, and *k*_cat_, in the absence of Ile were almost the same between the WT and the H480Y variant of Ilv1 (Table 1). When the kinetic parameters of the WT Ilv1 were measured in the presence of Ile, the *K*_0.5_ value for Thr was increased and the *V*_max_ and *k*_cat_ values were slightly decreased, resulting in 50% decrease in the catalytic activity. On the contrary, these kinetic parameters of the H480Y variant were not affected even in the presence of 10 mM Ile. Note that addition of Ile increased TD activity of the H480Y variant at the low concertation of Thr, leading to a decrease of the *K*_0.5_ value for Thr and a slight increase in the catalytic activity.

**TABLE 1:** Kinetic parameters of Ilv1^*a*^

Ilv1	Effector	*K*_0.5_	*V*_max_	*k*_cat_	*k*_cat_/*K*_0.5_
		(mM)	(U·min^−1^·mg^−1^)	(s^−1^)	(mM^−1^·s^−1^)
WT	None	40 ± 2.2	71 ± 3.9	75 ± 4.1	1.87
	0. 3 mM Ile	69 ± 19	62 ± 17	66 ± 18	0.95
H480Y	None	42 ± 2.4	71 ± 3.4	75 ± 3.6	1.78
	10 mM Ile	35 ± 3.9	65 ± 4.7	69 ± 4.9	1.96

a The values are the means and standard errors of results from three independent experiments.

### Effect of the His480Tyr substitution on the productivity of amino acid and fusel alcohol.

**FIG 4:**
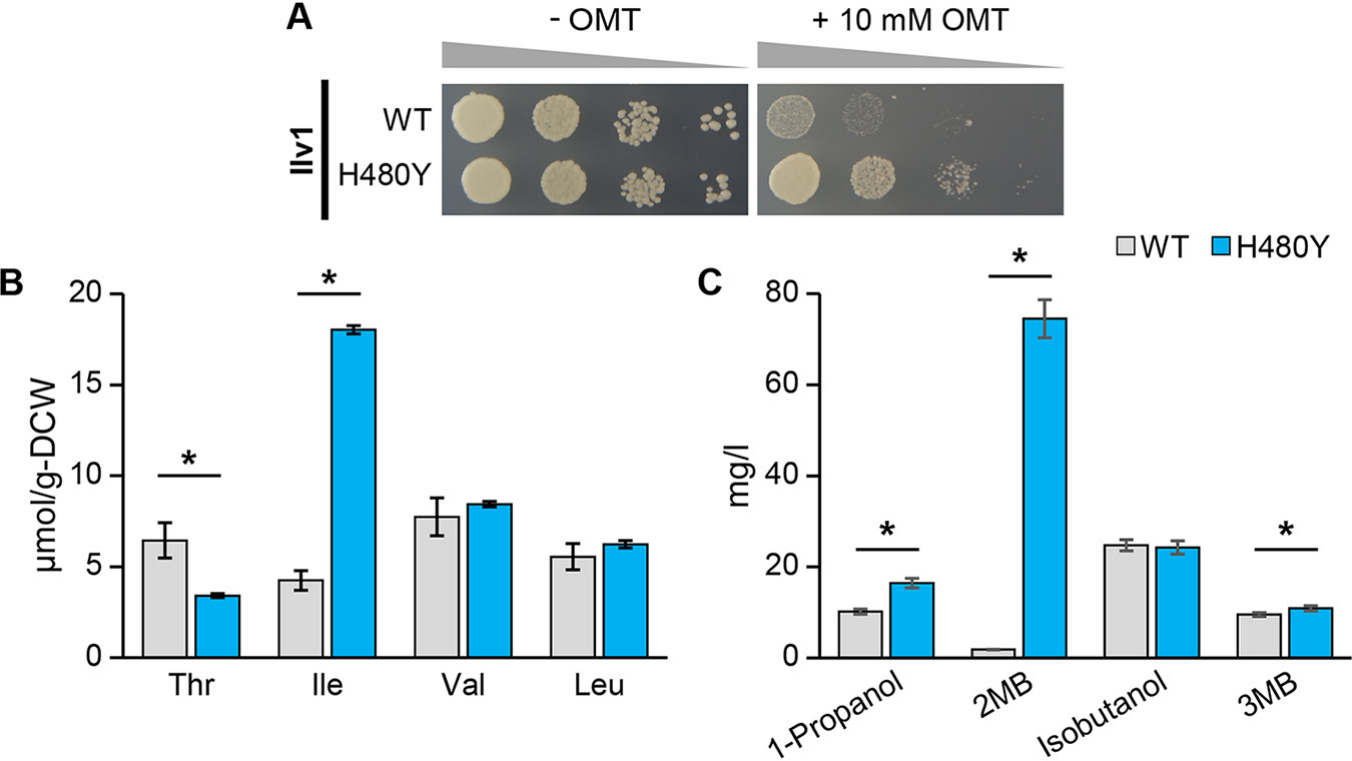
Characteristics of yeast cell expressing *ILV1*^H480Y^. (A) Sensitivity to OMT. *ilv1*Δ cells harboring *ILV1*^WT^ or *ILV1*^H480Y^ were cultured to the early log phase and spotted onto SD+Alt without OMT (left panel) or the same medium containing 10 mM OMT (right panel). (B and C) Intracellular amino acids content (B) and extracellular fusel alcohols content (C) in strains *ilv1*Δ harboring *ILV1*^WT^ (WT) or *ILV1*^H480Y^ (H480Y). Yeast cells were cultured in SD+Am medium, separated to cells and culture broth after 48 h cultivation, measuring amino acid contents in the cell and fusel alcohol contents in culture broth. 2MB: 2-methyl-1-butanol, 3MB: 3-methyl-1-butanol. Data are presented as means ± standard deviation from four independent experiments. Asterisks indicate statistically significant differences between two strains (Student's *t* test, *P < 0.05).

In order to confirm that the removal of allosteric regulation by Ile contributes to the enhancement of Ile productivity, we constructed expression plasmids for the *ILV1*^WT^ or *ILV1*^H480Y^ genes and expressed both genes in an *ILV1*-deleted laboratory strain of S. cerevisiae (*ilv1*Δ). As shown Fig. 4A, the *ilv1*Δ cells expressing *ILV1*^WT^ were sensitive to 10 mM OMT, whereas the expression of *ILV1*^H480Y^ conferred resistance to OMT on *ilv1*Δ cells, suggesting that the H480Y variant enhanced productivity of Ile in yeast cells. To further analyze the effect of the His480Tyr substitution on the productivity of amino acid and fusel alcohol, we cultivated *ilv1*Δ cells expressing the *ILV1*^WT^ or *ILV1*^H480Y^ gene in SD+Am medium, and quantitated the contents of intracellular amino acids and extracellular fusel alcohols (Fig. 4B and C). Yeast cells expressing *ILV1*^H480Y^ showed 4.2-fold increase in intracellular Ile content (18 μmol/g-DCW) compared to *ILV1*^WT^ (4.3 μmol/g-DCW) (Fig. 4B). Interestingly, in correspondence with an increase in the intracellular Ile content, the extracellular 2-MB content of *ilv1*Δ cells harboring *ILV1*^H480Y^ (75 mg/liter) was markedly increased compared with that (1.9 mg/liter) in the culture medium of *ilv1Δ* cells carrying *ILV1*^WT^ (Fig. 4C). Moreover, the expression of *ILV1*^H480Y^ decreased the intracellular Thr content but increased the extracellular 1-propanol level compared with those of *ilv1*Δ cells harboring *ILV1*^WT^ (Fig. 4B and C).

### Brewing characteristics of sake yeast mutant with intracellular Ile accumulation.

**FIG 5:**
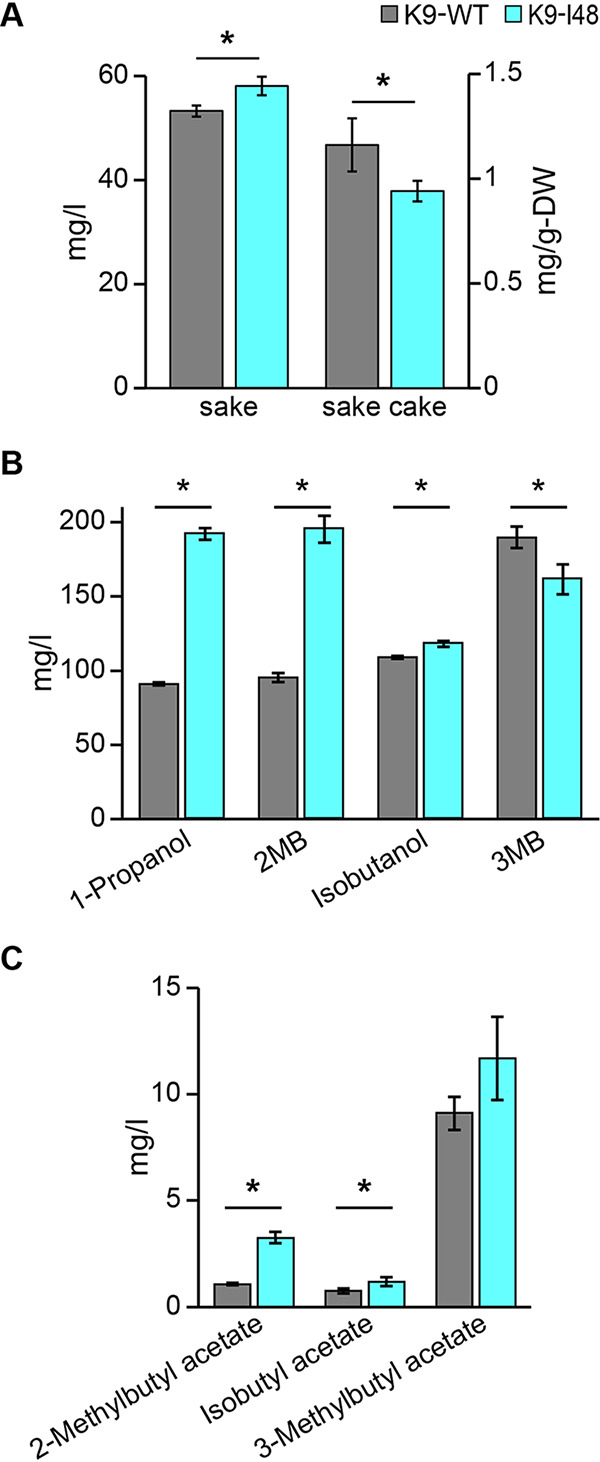
Characteristics of sake and sake cake brewed by K9-I48. (A) Ile contents in sake and sake cake brewed with strains K9-WT and K9-I48 (B and C) Fusel alcohol (B) and acetate ester of fusel alcohol contents (C) in sake brewed with strains K9-WT and K9-I48. 2-methylbutyl acetate, isobutyl acetate and 3-methylbutyl acetate are acetate esters corresponding to 2-methyl-1-butanol (2MB), isobutanol and 3-methyl-1-butanol (3MB), respectively. Data are presented as means ± standard deviation from three independent experiments. Asterisks indicate statistically significant differences between two strains (Student's *t* test, *P < 0.05).

Finally, we conducted a small-scale sake brewing test using strains K9-WT and K9-I48. No significant difference in CO_2_ emission (data not shown) or the general properties of sake, such as ethanol content (17.9% for K9-WT and 17.6% for K9-I48), acidity (2.4 for K9-WT and 2.3 for K9-I48) and amino acidity (1.7 for K9-WT and 1.8 for K9-I48), were found between sakes brewed with K9-WT and K9-I48. The Ile contents in sake and sake cake brewed with K9-I48 were slightly higher and lower than in those brewed with K9-WT (Fig. 5A). In contrast, the concentrations of 2MB and 2-methylbutyl acetate (an acetate ester of 2MB) in sake brewed with K9-I48 (195 mg/liter and 3.3 mg/liter) were approximately 2.1- and 3.0-fold higher than those in sake brewed with K9-WT (95 mg/liter and 1.1 mg/liter), respectively (Fig. 5B and C). Furthermore, the 1-propanol content (192 mg/liter) in sake brewed with K9-I48 was 2.1-fold higher than that of K9-WT (91 mg/liter), whereas almost the same amounts of 3MB, isobutanol and their acetate esters were observed between sakes brewed with strains K9-WT and K9-I48 (Fig. 5B and C).

## DISCUSSION

Here, we isolated a sake yeast mutant with Ile accumulation (strain K9-I48) and analyzed the mutant *ILV1* gene (*ILV1*^H480Y^) found in K9-I48. The intracellular Ile of K9-I48 cells cultivated in YPD rich medium (data not shown) was decreased compared with that in SD+Am medium (Fig. 2B), suggesting that K9-I48 does not carry mutation(s) for enhancement of Ile uptake. In addition, the flavor contents in sakes brewed with strains K9-WT and K9-I48 suggest that K9-I48 does not possess mutation(s) of the gene(s) in the Ehrlich pathway, which contribute to an increase in 2MB and 1-propanol in sake brewed with K9-I48, because there was little difference in contents of isobutanol, 3MB, and their acetate esters. We thus concluded that the His480Tyr substitution in Ilv1 is mainly responsible for high-level production of Ile in yeast cells. Desensitization of the H480Y variant to allosteric inhibition by Ile could allow accumulation of 2-oxobutyrate, the product of the reaction catalyzed by TD, in yeast cells. Excess 2-oxobutyrate is converted into Ile, 2MB and 1-propanol, leading to overproduction of these compounds (Fig. 4 and 5). The expression of the H480Y variant reduced intracellular Thr as shown in Fig. 4B; however, the Thr content in K9-I48 was almost the same as that in K9-WT (Fig. 2B). The different phenotypes between strains K9-I48 and *ilv1Δ* cells expressing *ILV1*^H480Y^ may be caused by other gene mutation(s) in strain K9-I48, which was isolated by random mutagenesis with EMS treatment. To explore this possibility, whole-genome sequence analysis of K9-I48 should be performed.

**FIG 6:**
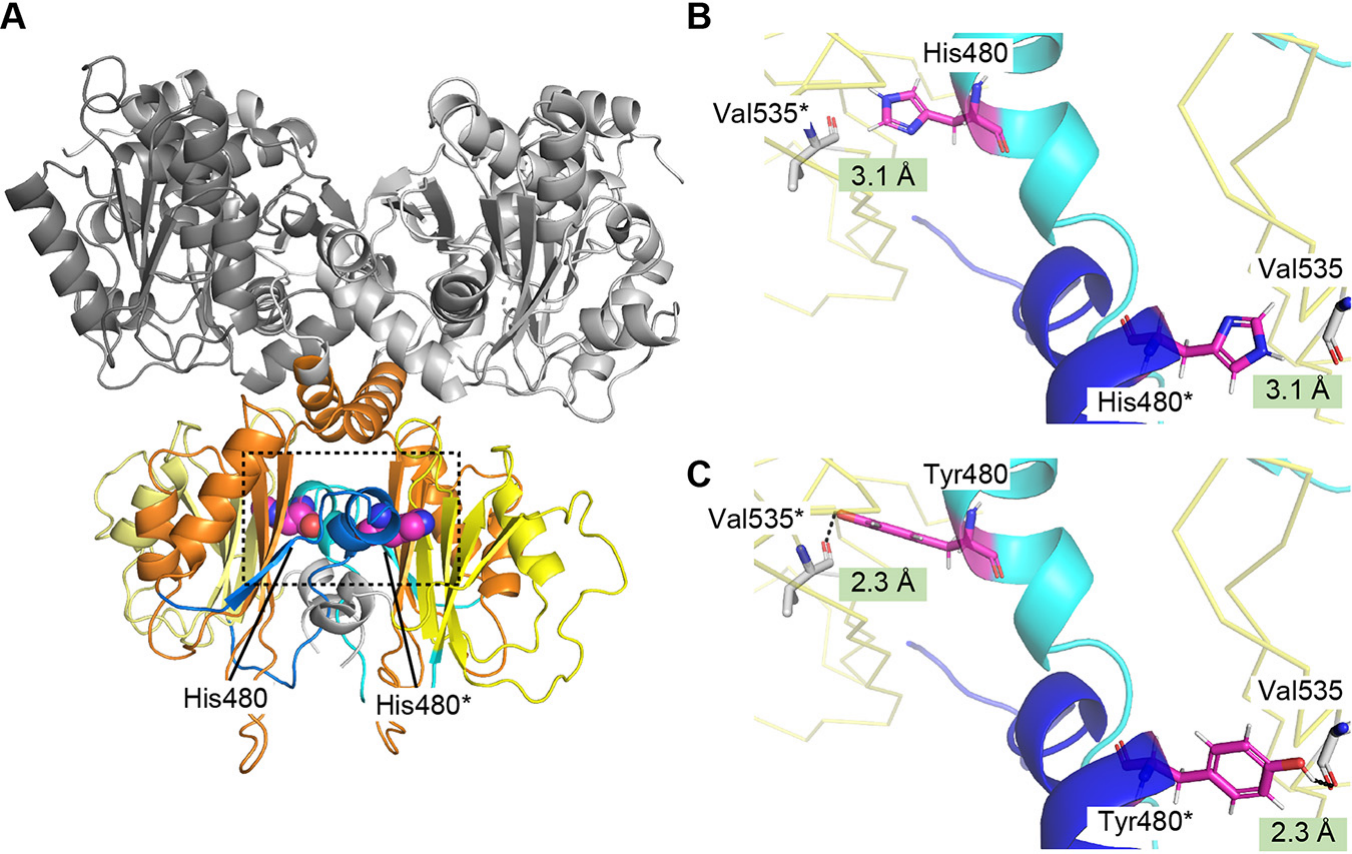
Structural comparison of the WT Ilv1 with the His480Tyr variant. (A) Homodimeric structure of Ilv1 homology model. Monomers of Ilv1 cross each other in homodimer. The contacts of two monomers are mainly occurred between the C-terminal domains, especially the linker region of one monomer located in the vicinity to that of another monomer. The whole protein structure is shown by a cartoon model. The catalytic domain, ACT-1, the linker region and ACT-2 are represented in gray, orange, blue and yellow, respectively. His480 is shown as a sphere model and in magenta. (B and C) Local structure around the residue at position 480 of the WT Ilv1 (B) and the His480Tyr variant (C). The ACT-2 domain is shown in yellow ribbon. The liker region of monomer A and B are represented as a carton model in cyan and blue, respectively. The residues at position 480 (His480 and Tyr480 in [B] and [C], respectively) and Val535 are shown as a stick model in magenta and white, respectively. Values in the green squares in panel B indicate the distance from the hydrogen of N-1 of the imidazole ring in the side chain of His480 to the oxygen of the carbonyl group in the main chain of Val535, whereas those in panel C represents the distance from the hydrogen of the hydroxyl group on C4 of the aromatic ring in the side chain of Tyr480 to the oxygen of the carbonyl group in the main chain of Val535. The predicted inter-monomer hydrogen bonds between Tyr480 and Val535 are represented black dashed lines. The asterisk indicates residues from monomer B. Panels (B) and (C) show a zoom up of local structure around the residue at position 480 (indicated by dashed square in [A]).

The enzymatic analysis revealed that the H480Y variant was insensitive to upregulation of enzymatic activity by Val as well as downregulation by Ile (Fig. 3). As previously reported in TDs from E. coli and *A. thaliana* (19, 38), addition of Ile altered the kinetic parameters of the WT enzyme, especially increase in the *K*_0.5_ value for Thr. One possibility is that binding of Ile may induce conformational changes of Ilv1, leading to a decrease in catalytic ability as reported in regulation of enzymes containing the ACT domain (24–26). Moreover, TD activity of the H480Y variant at the low concertation of Thr was increased in the presence of 10 mM Ile. Although a low concentration of Ile activated the enzymatic activities of TDs from S. cerevisiae and *A. thaliana*, its high concentration inhibited them (19, 37). These results suggest that the histidine to tyrosine substitution at position 480 affects the Ile-mediated conformational changes in allosteric inhibition by Ile. To date, the mechanisms in allosteric regulation with Ile and Val, however, have never been directly characterized on structural grounds. His480 is located in the linker region between two ACT-like domains (Blue bar in Fig. 1B) and far from effector binding sites; however, this histidine residue is highly conserved in various TDs, suggesting the importance of the histidine residue at position 480 (Fig. S1). Furthermore, Arg482 in the same region is conserved in basic amino acids (Fig. S1), and the substitution of corresponding arginine in the *A. thaliana* TD (Arg499) with cysteine desensitized to Ile feedback inhibition and increased Ile productivity, indicating that the linker region is involved in allosteric regulation (29). The crystal structure of the E. coli TD bound with the cofactor PLP revealed that the linker region is located at the interface of two monomers and forms the hydrogen bond networks within the monomers (33). We therefore hypothesized that His480 in the linker region may be involved in allosteric regulation through interactions within the oligomeric structure, resulting in incendivity to Ile-mediated feedback inhibition. In the homodimeric structure model of Ilv1, the linker region is located in the monomer-monomer interface, and His480 does not form any interactions with the neighboring monomer (Fig. 6A and B). In contrast, the histidine-to-tyrosine replacement at position 480 would cause the formation of a hydrogen bond between the hydroxy group in the phenolic side chin of Tyr480 and the carbonyl group in the main chain of Val535 in the ACT-like domain (ACT-2 in Fig. 1B) from another monomer (Fig. 6C). Although conformational changes in allosteric regulation of TD have been unclear due to the unavailability of the structural basis, it is possible that the inter-monomer hydrogen-bond formation in the H480Y variant contributes to the stabilization of the TD homodimer and prevents conformational (or oligomeric) alteration by Ile binding such as the oligomeric state change of the *A. thaliana* TD by Ile (27). On the other hand, Arg482 is located close to His480 in the linker region, the role of this arginine in allosteric regulation of Ilv1 may be different from that of His480. The side chain of Arg482 faces to ACT-1 in the same monomer and forms the hydrogen bonds with the side chain of Glu392 and Glu420. The cysteine replacement of this arginine, which was reported in *A. thaliana* TD, may cause disruption of the intra-monomer interactions between the linker region and the ACT-1 domain, leading to alteration in local conformation and binding affinity to inhibitor Ile. Further biochemical and structural analyses should clarify the role of the linker region between the ACT-like domains in the allosteric regulation of Ilv1, leading to elucidation of the detailed mechanism of allosteric regulation of various TDs.

In terms of biotechnological applications, increasing the Ile-derived volatile components of 2MB and its acetate ester, in sake brewed with K9-I48 is a promising approach to create new, distinctive sake flavors. However, the flavor contents in sake brewed with K9-I48 are similar to those of the previously obtained OMT-resistant yeast mutant which may also carry the feedback-inhibition insensitive variant of Ilv1 (39). The limited availability of precursors for Ile, such as aspartate and threonine, in yeast cells could produce almost the same amount of Ile and Ile-derived flavors during sake fermentation, leading to the similar flavor contents in sakes brewed with K9-I48 and the previously obtained OMT-resistant yeast mutant. In order to brewing sake with distinct taste and flavor, it is desirable to further increase the productivity of Ile-derived flavor compounds, such as fusel alcohols. Thus, the breeding of the yeast strains with excess precursors for Ile to increase the intracellular Ile content (40) will be a feature challenge.

In conclusion, we successfully isolated a diploid sake yeast mutant K9-I48 that overproduced not only Ile but also 2MB by a conventional mutagenesis technique. Strain K9-I48 possessed a novel mutation in the *ILV1* gene encoding the His480Tyr variant of TD. The His480Tyr substitution desensitized Ile feedback inhibition of TD activity, leading to high-level production of Ile and 2MB in yeast cells. The present study is the first to demonstrate that the conserved histidine residue located in the linker region between two regulatory ACT-like domains is involved in allosteric regulation of Ilv1. Moreover, sake brewed with K9-I48 contained 2–3 times more fusel alcohols than sake brewed with the parent strain. These results will contribute to the development of superior industrial yeast strains for high-level production of Ile and its related fusel alcohols.

## MATERIALS AND METHODS

### Strains and culture media.

The diploid sake yeast strain Kyokai No.9 (K9-WT, *MAT*a/α) and the haploid laboratory yeast strain BY4741/*ilv1*Δ (*MAT*a *his*3Δ1 *leu*2Δ0 *met*15Δ0 *ura*3Δ0 *ilv1*::*KanMX4*) (Horizon Discovery, Cambridge, UK) of S. cerevisiae were used as a parent strain for conventional mutagenesis and as a host strain for expression of the *IlV1* gene, respectively. A nutrient rich medium YPD (10 g/liter yeast extract, 20 g/liter peptone and 20 g/liter glucose) and a synthetic dextrose minimal medium SD+Am (1.7 g/liter yeast nitrogen base without amino acid and ammonium sulfate [Difco Laboratories, Detroit, MI, USA], 20 g/liter glucose and 5 g/liter ammonium sulfate) were used for cultivation of yeast cells. For isolation of OMT-resistant sake yeast mutants, yeast cells of strain K9-WT were grown in SD+Alt medium (SD medium supplemented with 5 g/liter allantoin as a nitrogen source instead of ammonium sulfate).

E. coli strains DH5α [F^–^ λ^–^ Φ80*lacZ*ΔM15 Δ(*lacZYA-argF*)*U169 deoR recA1 endA1 hsdR17*(r_K_^–^ m_K_^+^) *supE44 thi-1 gyrA96*] and BL21(DE3) [F^–^ *ompT hsdS*(r_B_^–^ m_B_^–^) *gal dcm λ*(DE3) (λ(DE3):*lacI*, *lacUV5-T7 gene1 ind1 sam7 nin5*] were used for construction of the plasmid and for expression of the recombinant Ilv1, respectively. E. coli strains were cultured in Luria-Bertani (LB) medium (5 g/liter yeast extract, 10 g/liter tryptone and 5 g/liter NaCl) or TB medium (12 g/liter yeast extract, 24 g/liter tryptone, 5.02 g/liter glycerol, 170 mM KH_2_PO_4_ and 720 mM K_2_HPO_4_) containing appropriate antibiotics.

### Construction of expression plasmids for the *ILV1* genes.

The DNA fragment, including 1,000 bp upstream and downstream of the open reading frame (ORF) of *ILV1,* was amplified from the genomic DNA of strain K9-WT by PCR with the primers (5′-C GAC TCT AGA GGA TCC CCT GTC ACA CCC GCT GTA TCA AAG-3′ and 5′-AT GAT TAC GAA TTC GAG CTC TCA CCG CCA ATG TCA CCG CT-3′, the underlined nucleotides indicate the *Bam*HI and *Sa*cI recognition sites). The PCR-amplified DNA fragment was sub-cloned into the *Bam*HI-*Sa*cI site of vector pUC18 by In-Fusion HD Cloning kit (TaKaRa Bio, Shiga, Japan), resulting in pUC18_*ILV1*. The point mutation (C1438T) was introduced into the *ILV1* gene on pUC18 with the primers (5′-GAA TTG GCT AAA TCT T*AT GGT AGA TAC TTG G-3′ and 5′- CCA AGT ATC TAC CAT A*AG ATT TAG CCA ATT C-3′, the asterisks indicate the position of the nucleotide mutation), leading to His480Tyr substitution on Ilv1, pUC18_*ILV1*^H480Y^. In order to construct the expression plasmids for the *ILV1* gene in yeast, the DNA fragments, including upstream and downstream of the *ILV1* ORF, were obtained by digesting pUC18_ *ILV1* and pUC18_*ILV1*^H480Y^ with *Bam*HI-*Sa*cI and then ligated into the same site of vector pRS416, resulting in pRS416_*ILV1* and pRS416_*ILV1*^H480Y^.

In order to construct the expression plasmids for the recombinant Ilv1 proteins, we amplified the *ILV1* gene with lack of nucleotides corresponding to the N-terminal 45 amino acid residues as the mitochondrial localization signal from pUC18_ *ILV1* and pUC18_*ILV1*^H480Y^ with primers (5′-GGG GAC AAG TTT GTA CAA AAA AGC AGG CTT ACA CTC TGA ATT GAA ATT GGA TG-3′ and 5′- GGG GAC CAC TTT GTA CAA GAA AGC TGG GTG ATA TTT CAA GAA TTT TTG ATA AAC-3′) (32). The amplified DNA fragments were introduced into vector pDONR221 (Thermo Scientific, Waltham, MA, USA) using BP clonase II (Thermo Scientific), resulting in pDONR_*ILV1* and pDONR_*ILV1*^H480Y^. The *ILV1* genes were then transferred to the pET53-dest expression vector (Thermo Scientific) using LR clonase II (Thermo Scientific), resulting in pET_*ILV1* and pET_*ILV1*^H480Y^.

### Isolation of OMT-resistant sake yeast mutants.

Strain K9-WT was randomly mutagenized by treatment with 5.5% of EMS in phosphate buffer (pH 7.0) at 30°C for 60 min. EMS treated cells were washed by 10% (wt/vol) sodium thiosulfate twice and then spread onto SD+Alt medium containing 10 mM OMT. After incubation at 30°C for 2–3 days, the resulting colonies were collected and tested for OMT-resistance and amino acid production. The survival rate of yeast cells after EMS treatment was around 20–40%.

### Spot test for OMT-resistance of yeast strains.

Yeast cells were cultured in SD+Am medium and then inoculated into new medium at a final optical density 600 nm (OD_600_) of 0.03. After cultivation to reach an OD_600_ of 1.0 at 30°C, yeast cells were collected and washed by sterilized water twice. Serially diluted yeast cells were spotted onto SD+Alt plates in the absence or in the presence of OMT. The plates were incubated for 2 days at 30°C.

### Quantification of intracellular amino acids content.

Yeast cells were cultured in SD+Am medium for 2 days at 30°C, then inoculated into the same medium at an OD_600_ of 0.1. After cultivation for 48 h at 30°C under shaking, the yeast cells were harvested by centrifugation and washed twice. The resulting yeast pellet was used for measurement of intracellular amino acid contents. In the case of yeast cells expressing *ILV1*^WT^ and *ILV1*^H480Y^, the separated supernatant was used for quantification of extracellular fusel alcohol contents (in “Quantification of extracellular fusel alcohols content”). Next, harvested cells were resuspended in sterilized water and the suspension was adjusted to an OD_600_ = 40. Consequently, amino acids in an aliquot (0.5 ml) of the cell suspension were extracted by boiling water at 100°C for 20 min. After centrifugation (5 min at 15,000 × *g*), the amino acid content in each supernatant was subsequently quantified with an amino acid analyzer with ion-exchange chromatography and post-column ninhydrin derivatization (JLC-500/V2, JEOL, Tokyo, Japan). The content of each amino acid was represented as μmol per g dry cell weight (DCW).

### Quantification of extracellular fusel alcohols content.

Fusel alcohols content in the supernatant of yeast culture was quantified by using a gas chromatography (GC) as described previously (7) with slight modifications. 1.8 ml of the supernatant was mixed with 0.2 ml of internal standard (0.5 mg/ml of n-amyl alcohol) for quantification in the internal standard method. GC/MS analysis of the flavor components was conducted a GCMS-QP2010 SE (Shimadzu, Kyoto, Japan) equipped with an AOC-5000 PLUS autosampler (Shimadzu) and a DB-5MS column (30 m x 0.25 mm i.d., film thickness 0.25 μm; Agilent Technologies, Santa Clara, CA, USA) under the following chromatographic conditions: column temperature, 30°C for 7 min, and increased to 250°C at a rate of 20°C/min, and was maintained at 250°C for 5 min; ionization energy, 70 eV; ion source temperature, 200°C; interface temperature, 250°C; carrier gas, helium; flow-rate, 1.28 ml/min. The sample solutions were treated at 70°C for 30 min and then the gas phase was injected into GC/MS system in the split mode (split ratio, 1:5). The amount of flavor components (2MB, 3MB, isobutanol and 1-propanol) were calculated from a standard curve, which was obtained from the peak area of authentic reference samples using selected ion monitoring (SIM) mode: 2MB (*m/z*: 57, 70), 3MB (*m/z*: 55, 70), isobutanol (*m/z*: 31, 74), 1-propanol (*m/z*: 31, 59) and n-amyl alcohol (*m/z*: 55, 70).

### Expression and purification of the recombinant Ilv1 proteins.

E. coli BL21(DE3) harboring plasmid pET_*ILV1* and pET_*ILV1*^H480Y^ were cultivated in TB medium containing ampicillin and grown at 37°C to an OD_600_ of 1.5. The cells were cooled on ice for 5 min and isopropyl β-d-1-thiogalactopyranoside was added to a final concentration of 0.2 mM. After 20 h of cultivation at 18°C, the cells were harvested by centrifugation and suspended in buffer A [50 mM potassium phosphate (pH 8.0), 500 mM NaCl, 1 mM tris(2-carboxyethyl)-phosphine hydrochloride and 10% (wt/vol) glycerol]. The cell suspension was homogenized under cooling and then centrifuged to remove insoluble fraction. The supernatant was filtrated by 0.45 μm filter and subsequently applied onto a nickel affinity column (Ni Sepharose^TM^ 6 Fast fljow; GE Healthcare, Chicago, IL, USA). After the column was washed with buffer A containing 40 mM imidazole, the recombinant proteins were eluted by buffer A supplemented with 500 mM imidazole. The elution fraction was dialyzed with buffer containing 50 mM potassium phosphate (pH 8.0), 200 mM NaCl, 1 mM dl-dithiothreitol and 10% (wt/vol) glycerol at 4°C.

### Assay of TD activity of the recombinant Ilv1 proteins.

TD activity was measured by the production of ammonium in an enzyme-coupled system with glutamate dehydrogenase (GDH). The reaction mixture (final volume, 1 ml) contained the following: 100 mM potassium phosphate (pH 8.0), 50 mM Thr, 0.05 mM pyridoxal-5-phosphate, 6 mM 2-oxoglutarate, 0.25 mM NADH, 20 U of GDH (Toyobo, Osaka, Japan), 1.5 μg of purified Ilv1 and various concentrations of Ile or Val. The reaction mixture except for Thr was pre-equilibrated for 3 min at 30°C, and then the reaction was initiated by the addition of Thr. Ilv1-dependent oxidation of NADH by GDH was monitored at 340 nm with a DU-800 spectrophotometer (Beckman Coulter, Brea, CA, USA) and maintained at 30°C. In order to examine the allosteric regulation of Ilv1 by Ile, Ile was added to the reaction mixture at a concentration of 0–1.0 mM for the wild-type (WT) and 0–10 mM for the H480Y variant Ilv1, respectively. Whereas, for determination of the effect by Val, 0-5 mM Val was added to the reaction context. For steady-state kinetics, the concentrations of Thr were varied (20-105 mM for WT and 15–105 mM for the H480Y variant). In order to examine the influence of Ile on the steady-state kinetic parameter of TDs, the concentration of Ile was fixed at 0.3 mM for WT and 10 mM for the H480Y variant, and the concentrations of Thr were varied the same as above. The reaction rate was calculated with the extinction coefficient of NADH, 6,220 M^−1^cm^−1^. One unit of activity was defined as the amount of enzyme required to produce 1 μmol of ammonium per min. Kinetic parameters of each enzyme were calculated with GraphPad Prism version 9 (GraphPad Software) using nonlinear regression analysis.

### Small-scale sake brewing test with sake yeast strains.

Sake brewing test was carried out according to the previous report (41) with modification. Briefly, sake mash contained 29 g of pre-gelatinized rice with a polishing ratio of 70%, 7 g of dry koji (A. oryzae was grown on steamed rice), 150 μl of 9% (vol/vol) lactic acid, 71 ml of water and 1 × 10^9^ sake yeast cells. Fermentation was conducted at 15°C and was monitored by measuring the volume of evolved CO_2_ using Fermograph II (Atto, Tokyo, Japan). After 14 days of fermentation, the sake mash was centrifuged and supernatant was obtained as sake. The general properties of sake were analyzed in accordance with the standard method established by the National Tax Administration Agency.

Amino acid contents in sake cake and sake were quantified as same method as described in “Quantification of intracellular amino acids content” after following pretreatments. Approximately 200 mg of sake cake (wet weight) was suspended in 1.5 ml of sterilized water and then amino acids in sake cake were extracted by boiling water at 100°C for 30 min. After removal of debris by centrifugation, the supernatant was separated as an extract of sake cake. The extracts of sake cake and sake were 5- and 10-folds diluted, respectively, and analyzed with an amino acid analyzer. The amount of amino acid content in sake and sake cake were presented as mg per liter and μmol per g dry weight. Flavor contents in sake was separated by the same GC method as described in “Quantification of extracellular fusel alcohols content,” and quantified with a flame ionization detector or MS detector.

### Structural analysis of the Ilv1 protein.

The homology model of Ilv1 (the S. cerevisiae TD) was constructed using the Swiss-model (42) with the crystal structure of the E. coli TD (48% sequence identity to Ilv1, Protein Data Bank ID: 1TDJ [33]) as a template. The amino acid substitution of histidine to tyrosine at position 480 was introduced using PyMOL software (the PyMOL Molecular Graphics System version 2.5; Schrödinger, LLC). The structure model of the WT and His480Tyr variant Ilv1 were drawn using PyMOL software (http://www.pymol.org).

### Data availability.

The data underlying this article are available in the article.
