# Supplementary material for: High-Level Production of Isoleucine and Fusel Alcohol by Expression of the Feedback Inhibition-Insensitive Threonine Deaminase in Saccharomyces cerevisiae
Source: Appl Environ Microbiol. 2022 Mar 8;88(5):e02130-21. doi: 10.1128/aem.02130-21 (PMC8904041; doi:10.1128/aem.02130-21)
Supplement: Supplemental file 1 — Fig. S1 to S3. Download aem.02130-21-s0001.pdf, PDF file, 0.8 MB [file aem.02130-21-s0001.pdf]

Supplemental Material

High-level Production of Isoleucine and Fusel alcohol by expression of the Feedback Inhibition-insensitive Threonine deaminase in *Saccharomyces cerevisiae*

Shota Isogai[a], Akira Nishimura[a], Atsushi Kotaka[b], Naoyuki Murakami[b], Natsuki Hotta[b], Hiroki Ishida[b], Hiroshi Takagi[a#]

[a] Division of Biological Science, Graduate School of Science and Technology, Nara Institute of Science and Technology, 8916-5 Takayama, Ikoma, Nara 630-0192, Japan

[b] Research Institute, Gekkeikan Sake Co. Ltd., 101 Shimotoba-koyanagi-cho, Fushimi-ku, Kyoto 612-8385, Japan

Running title: Isoleucine and fusel alcohol production in Yeast

#Address correspondence to Hiroshi Takagi, hiro@bs.naist.jp

This file includes FIG S1, S2, and S3.

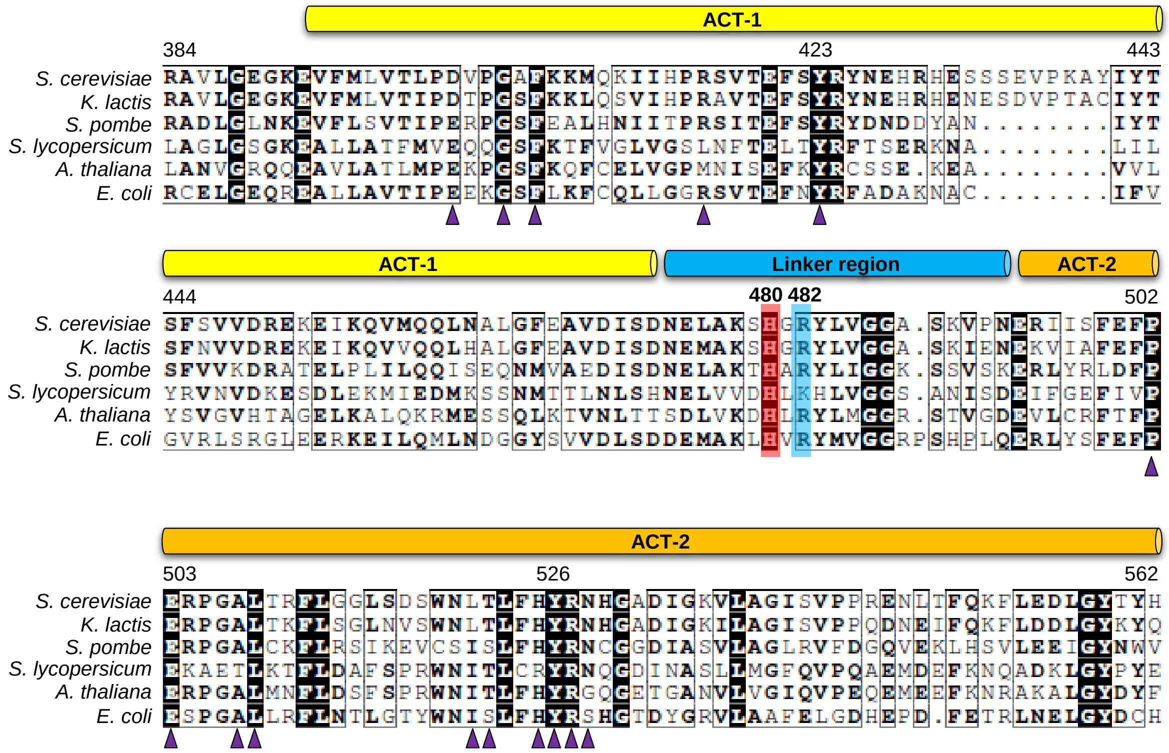

FIG S1 Comparison among biosynthetic TDs. Sequence alignment of the C-terminal region of Ilv1 among various microorganisms and plants. The amino acid sequence of the *S. cerevisiae* Ilv1 (Genbank ID code: NP_011009) was compared to those of *Kluyveromyces lactis* (QEU61378), *Schizosaccharomyces pombe* (NP_596641), *Solanum lycopersicum* (NP_001296095), *Arabidopsis thaliana* (NP_187616) and *Escherichia coli* (NP_418220) homologues. Numbering of residues is in Ilv1 and conserved residues were highlighted in black boxes. His480 andArg482 are shown in red and light blue, respectively. The residues involved in allosteric regulation are represented by purple triangles. Yellow, blue and orange bars above the alignment represented the domain organization of Ilv1 as described in Fig. 1B.

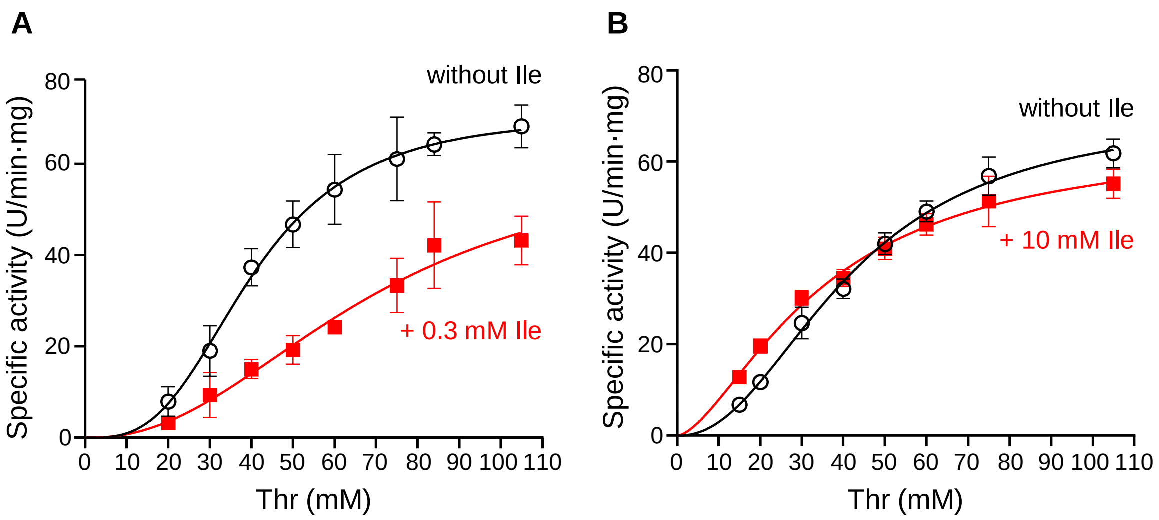

FIG S2 Effect of Ile on steady-state kinetics of the WT (A) and the H480Y variant (B) of Ilv1. TD activities of the recombinant WT (A) and H480Y variant (B) enzymes were measured in the absence (black open circles) or in the presence of Ile (red filled squares). Data are presented as means ± standard deviation from three independent experiments. For the WT enzyme, the concentration of Ile was fixed 0.3 mM, which is close to the half maximal inhibitory concentration (IC_{50}) value determined from Fig. 3A. Whereas, 10 mM of Ile was used for the H480Y variant.

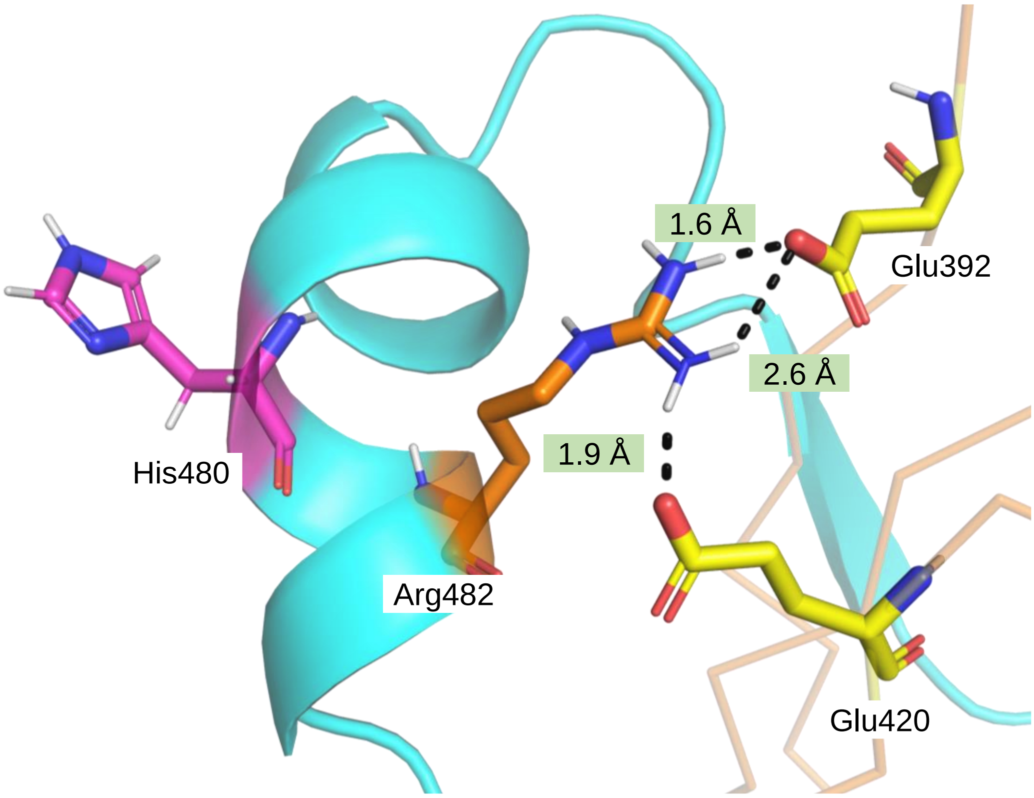

FIG S3 Intra-monomer interactions between Arg482 and the residues in the ACT-1 domain. Local structure around the linker region of the WT Ilv1 was shown. The liker region is represented as a carton model in cyan. The His480, Arg482, Glu392, and Glu 420 are shown as a stick model in magenta, orange, and yellow, respectively. The ACT-1 domain is shown in orange ribbon. The predicted intra-monomer hydrogen bonds among Arg482, Glu392, and Glu420 are represented black dashed lines. Values in the green squares indicate the distance between atoms which form the hydrogen bonds.
